# An Energy Efficient Distance-Aware Routing Algorithm with Multiple Mobile Sinks for Wireless Sensor Networks

**DOI:** 10.3390/s140815163

**Published:** 2014-08-18

**Authors:** Jin Wang, Bin Li, Feng Xia, Chang-Seob Kim, Jeong-Uk Kim

**Affiliations:** 1 College of Information Engineering, Yangzhou University, Yangzhou 225009, China; E-Mails: wangjin@yzu.edu.cn (J.W.); lb@yzu.edu.cn (B.L.); 2 School of Software, Dalian University of Technology, Dalian 116620, China; E-Mail: f.xia@ieee.org; 3 Department of Energy IT, Gachon University, Seongnam 461-701, Korea; E-Mail: cskim407185@gmail.com; 4 Department of Energy Grid, Sangmyung University, Seoul 110-743, Korea

**Keywords:** wireless sensor networks, mobile sink, distance aware, energy, lifetime

## Abstract

Traffic patterns in wireless sensor networks (WSNs) usually follow a many-to-one model. Sensor nodes close to static sinks will deplete their limited energy more rapidly than other sensors, since they will have more data to forward during multihop transmission. This will cause network partition, isolated nodes and much shortened network lifetime. Thus, how to balance energy consumption for sensor nodes is an important research issue. In recent years, exploiting sink mobility technology in WSNs has attracted much research attention because it can not only improve energy efficiency, but prolong network lifetime. In this paper, we propose an energy efficient distance-aware routing algorithm with multiple mobile sink for WSNs, where sink nodes will move with a certain speed along the network boundary to collect monitored data. We study the influence of multiple mobile sink nodes on energy consumption and network lifetime, and we mainly focus on the selection of mobile sink node number and the selection of parking positions, as well as their impact on performance metrics above. We can see that both mobile sink node number and the selection of parking position have important influence on network performance. Simulation results show that our proposed routing algorithm has better performance than traditional routing ones in terms of energy consumption.

## 1. Introduction

Recent advances in wireless communication, computer technology and micro-electronics technology have enabled the rapid development of tiny, low-cost, and multi-functional sensor nodes. These sensor nodes can be randomly deployed in a certain area to sense, process and transmit their monitored data to some remote base station. In this way, wireless sensor networks (WSNs) are formed with large number of tiny sensor nodes which usually work in a local and collaboratively way. WSNs have broad application fields such as military surveillance, environment monitoring and forecast, structural monitoring and target tracking, home network and healthcare *etc.* [1–4].

Energy efficiency is a key research concern and challenging issue during the design of routing algorithms for WSNs. The autonomous sensor nodes are usually powered with very limited energy supply. Besides, the number of sensor nodes is usually large, and it is impractical to recharge or replace the battery power of sensor nodes. Moreover, long distance communication between sensor nodes and base station (or sink nodes) may also increase overall energy consumption. A well-known problem is that the sensors close to sink node will bear more traffic burden to forward to the sink nodes and they will deplete their energy much faster than those sensor nodes far away from sink nodes, leading to network disconnection and much shortened network lifetime. Those sensor nodes become bottleneck nodes and they are called hot spots, and this phenomenon is referred to as the energy holes problem. In the meantime, a large quantity of energy may not be used efficiently for the transmission of the rest of the data among many other residual sensors, which is not very desirable.

Exploiting sink mobility based technology is gaining more and more popularity in recent years to achieve better energy efficiency and lifetime performance. Many studies have shown that sink mobility technology can significantly balance traffic load and improve network performance [3,4]. Mobile sink nodes moving at a certain speed throughout a sensing field can collect monitored data from the static sensors in a single-hop or multi-hop transmission manner. In this way, one can effectively reduce energy overhead at sensor nodes near sink nodes, and enable the sensor network to last longer. As the mobile sink nodes change position, their neighbor nodes will also change. Intuitively, the introduction of sink mobility technology into WSNs can effectively alleviate the hot spot problem.

Another advantage of utilizing sink mobility technology is that, since the energy consumption will grow exponentially with the transmission range (typically with an exponent range between 2 and 4), energy consumption with mobile sink nodes can be greatly reduced by using a relatively short transmission distance on average, as sink nodes will choose nodes located at a short distance to communicate.

The main contribution of this paper lies in the following three aspects: First, we perform a detailed review of the literature about data collection mechanisms with multiple mobile sinks for wireless sensor networks, and contrast the main features of this research work. Second, we propose an energy efficient distance-aware routing algorithm with multiple mobile sinks for WSNs, which consists of route setup phase, steady phase and maintenance phase. Both distance and residual energy factors are considered during the routing phase, and sink nodes will move along the network boundary to collect monitored data. Finally, we perform an extensive simulation and analysis to validate the performance of our proposed algorithm in terms of energy consumption, network lifetime, *etc.*

The rest of the paper is organized as follows: Section 2 presents the literature survey, and Section 3 gives the system model which includes energy and network model. In Section 4, our proposed routing algorithm is explained in detail. Extensive simulation results are described and analyzed in Section 5, with further discussion presented in Section 6. Finally, Section 7 concludes this paper with some future research work.

## 2. Related Work

The introduction of mobile sinks in WSNs has gained more and more attention to balance energy consumption and prolong network lifetime. In 2005 and 2006, some predefined sink movement trajectories were studied. In [5], the authors investigated the benefits of a heterogeneous architecture for WSNs composed of a few resource-rich mobile nodes and a large number of simple static nodes. They studied WSNs with one mobile sink and one mobile relay individually. In [6], the authors studied a base station which moves along a predetermined movement path to collect data from cluster heads (CHs). Sensor nodes which were closest to mobile sink trajectory will be chosen as CHs, and CHs sent data to mobile node as it passed by. In [7], the authors proposed a hierarchical cluster-based data dissemination scheme called HCDD to disseminate data to mobile sinks with light control overhead. In HCDD, sensor nodes found the route without the location information of sensor nodes, and HCDD operated without using any expensive and power-consuming GPS device to estimate the location information. This was the first attempt to utilize single mobile sinks to achieve better network performance, and the sink moving strategies are mostly intuitive and simple.

In 2008, the optimal sink movement trajectory was further studied. In [8], the authors first explored and categorized the general problem of sink mobility in the context of trade-offs between data delivery delay and network lifetime. Then they studied a novel mobility control solution in which sensor nodes cooperatively determine the sink trajectory, and navigate mobile sinks for delay and energy optimized data collection. In [9], the authors proposed a data dissemination protocol and defined a vertical line that divides the sensor field into two equal parts. This line acted as a rendezvous area for data storage and look up. In [10], the authors focused on the problem of finding an optimal path of a mobile device to achieve the smallest data delivery latency in the case of minimum energy consumption at each sensor node. They demonstrated that their algorithm successfully finds the paths that result in 10%–50% shorter latency compared to previously proposed methods. During this stage, some early theoretical work to find optimal sink moving trajectories was performed with certain constraints. However, the authors also mainly dealt with single sink node based on their own devising scenarios, some of which were not feasible to practical application.

In 2010 and 2011, sink mobility technology drew much more attention with deeper study. In [11], the authors studied the backbone-based virtual infrastructure (BVI) to avoid the routing structure construction per each mobile sink by full network flooding. On that basis, they proposed a novel BVI-based communication protocol and showed that their proposed protocol was superior to the existing protocols in terms of the control overhead and the data delivery hop counts. In [12], the authors first investigated the optimum routing strategy for the static sensor network, and then further proposed a number of motions stratified for the mobile sinks to gather real time data from static sensor network, with the objective of maximizing the network lifetime. In particular, they considered a more realistic model where the moving speed and path for mobile sinks are constrained. In [13], the authors proposed a novel geographic routing for mobile sinks. The proposed scheme took advantage of the wireless broadcast transmission nature of sensor nodes. With the moving of the sink node, the new location information was propagated along the reverse geographic routing path to the source during data delivery. The authors proposed a data collection scheme, called the maximum amount shortest path (MASP) which increased network throughput and conserved energy by optimizing the assignment of sensor nodes [14]. MASP was formulated as an integer linear programming problem and solved based on genetic algorithm. In [15], the authors proposed a new data gathering scheme called SPAT to enable reliable and efficient data gathering by mobile sinks. This scheme guaranteed the mobile sink to collect data from all sensor nodes, and ensured fairness regarding to equally collecting data from the sensor nodes. During this stage, not only single mobile sink but also multiple mobile sinks based routing algorithms were proposed together with other techniques such as clustering, data aggregation *etc.* The joint problem of multiple mobile sinks and efficient routing is a more complex and challenging research issue, which was put forwarded without good solutions then.

Even though mobile sinks can improve network performance, they might also cause degraded network performance like long data collection delay, additional overhead of frequent location update *etc.* In recent years, the authors in [16] recently proposed an efficient data driven routing protocol (DDRP), which exploited the broadcast feature of wireless medium for route learning. The overhearing of transmission of data packet, which carries an additional option recording, will gratuitously provide each listener a route to a mobile sink. Random walk routing simply will be adopted for data packet forwarding, when no route to mobile sink is known. In [17], the authors first proposed a general mobility-assisted data collection (MADC) model which includes many important parameters such as number of mobile sinks, sink velocity, and traveling path. Then they developed a comprehensive theoretical approach to obtain achievable throughput capacity and lifetime. By applying the proposed approach, they investigated the behaviors of WSNs with one or more mobile sinks. In [18], the authors proposed a novel localized integrated location service and routing scheme, based on the geographic routing protocol GFG. In their scheme, sink updates location to neighboring sensor nodes after or before a link breaks. Considering both unpredictable and predictable sink mobility, the authors compared their scheme with an existing competing algorithm through simulation and validated that their scheme can generate routes close to shortest paths at dramatically lower message cost. In [19], the authors provided a simulation-based analysis of the energy efficiency of WSNs with static and mobile sinks where the focus was on two important configuration parameters: mobility path of the sink and duty cycling value of the nodes. The authors quantitatively analyzed the influence of duty cycling and the mobility radius of the sink as well as their interrelationship in terms of energy consumption for a well-defined model scenario. The analysis started from general load considerations and was refined into a geometrical model. This model was validated by simulations which are more realistic in terms of duty cycling than previous work. In [20], a survey about mobile sinks supported routing protocols for WSNs are presented, where the advantages and disadvantages of state-of-the-art routing protocols are categorized and discussed. During this stage, both the advantage and disadvantage of introducing multiple mobile sinks are more deeply studied with various constraints under different application scenarios. Joint optimization of sink moving strategy and routing algorithm becomes more challenging under more constrained conditions.

## 3. System Model

### 3.1. Basic Assumptions

We consider in this paper a wireless sensor network composed of a certain number of static sensor nodes and multiple mobile sink nodes. Each sensor node has a unique identifier (ID) that differs from other nodes. We make several basic assumptions, as follows:

(1) All sensor nodes are stationary after been deployed in the rectangular region.
(2) Wireless links are symmetric.
(3) Sensor nodes are location-aware.
(4) Each sensor node can adjust their transmission power based on relative distance.
(5) Sinks are energy-unconstrained and they can move and communicate anywhere in the network.

### 3.2. Network Model

**Figure 1.**
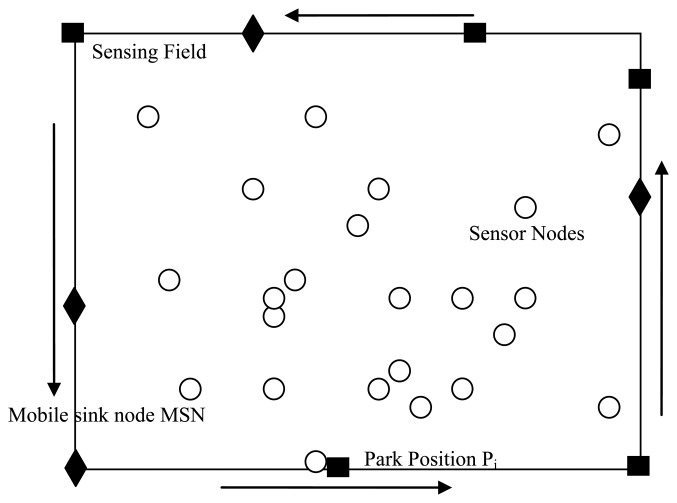
Network model.

A wireless sensor network can be viewed as a directed graph *G* = < *V_node_* U *V_sink_*, *E_node_* U *E_sink_* >, where *V_node_* and *V_sink_* represents the set of sensor nodes and sink nodes respectively. *E_node_* represents the set of all links *l(i,j)*, where *i* and *j* are neighboring sensor nodes, and *E_sink_* represents the set of all links between sensor nodes and sink node. The randomly distributed wireless sensor network is illustrated in Figure 1.

The sensor network here consists of a certain number of static sensor nodes as well as multiple mobile sink nodes which will move along the network boundary. The static sensor nodes are randomly dispersed in the monitored area and keep generating data packets. The mobile sink nodes are used to gather data packets in different location by moving along network boundary for two reasons. First, it is relatively easy to deploy mobile sink along network boundary rather than along certain optimal trajectory inside. More importantly, network performance is usually better with sink moving along the boundary than inside sensor network [19]. Here, the small squares *P_i_* stands for parking positions of mobile sink nodes.

Mobile sink nodes will periodically visit these parking positions and sojourn at each position for a certain time to collect data packets. We can divide the whole process of mobile sink node movement into a moving phase and a collecting phase. In the moving phase, mobile sink nodes only move with a certain speed from one parking position *P_i_* to another position *P_j_*, and they will refuse to receive any monitored data. In the collecting phase, mobile sink nodes will broadcast a notification message to their nearby sensors. The message includes their arrival message and next location message, and then they will receive data packets in a single hop or multi-hop transmission manner.

### 3.3. Energy Model

We use a simple energy model for the radio hardware energy dissipation to calculate the energy consumption, which is called the first radio energy model [2–4]. Depending on the distance between source node and destination node, a free space (*d^2^* power loss) or multipath fading (*d^4^* power loss) channel model will be used. To transmit an l-bit data packet over a distance *d*, *E_Tx_* amount of energy will be consumed as follows:

$$E_{Tx}(l,d)=\{\begin{array}{cc} lE_{\text{elec}}+l\varepsilon_{fs}d^{2}, & d<d_{0}\\ lE_{\text{elec}}+l\varepsilon_{mp}d^{4}, & d\geq d_{0} \end{array}$$

where *E_elec_* represents the energy dissipation of radio device. Its value is depended on many factors such as digital coding, modulation, and spreading of signal. *ε_fs_* and *ε_mp_* represents the free space and multipath transmitter amplifier model respectively. To receive the same data packet, *E_Rx_* amount of energy will be consumed with *E_Rx_(l)* = *lE_elec_*.

Depending on the distance from source to destination node, a threshold *d_0_* can be defined as $d_{0}=\sqrt{\varepsilon_{fs}/\varepsilon_{mp}}$, when we let $\varepsilon_{fs}d_{0}^{2}=\varepsilon_{mp}d_{0}^{4}$. If the distance to receiver is less than the threshold *d_0_*, the free space model is used; otherwise, the multipath model is used. Thus, to forward *k* data packets, *E_Fw_* amount of energy will be consumed, as is listed in Equation (1) below:

(1) $$E_{Fw}=\{\begin{array}{ll} (k+1)lE_{\text{elec}}+l\varepsilon_{fs}d^{2}, & d<d_{0}\\ (k+1)lE_{\text{elec}}+l\varepsilon_{mp}d^{4}, & d\geq d_{0} \end{array}$$

## 4. Our Proposed Routing Algorithm

Sink mobility strategies provide an efficient alternative to balance energy consumption and prolong the lifetime for WSNs. In most previous routing algorithms or protocols for static sensor networks, the routing tree is usually constructed by establishing explicit links between source and destination sensor nodes. Source nodes usually transmit their monitored data to their destination sensor node with single hop or multi-hop transmission manner. However, sensor nodes close to static sink nodes bear much heavier traffic loads than those away from the sink node. Thus, they will deplete their energy much more rapidly, resulting in the network performance degradation.

To achieve sink mobility, those mobile sink nodes can be mounted buses or other vehicles moving along fixed trajectories, or be fixed on robots, people or even animals. Here, we will describe our energy efficient distance-aware routing algorithm with multiple mobile sinks in a rectangle sensor network in detail.

### 4.1. Route Setup Phase

Mobile sink nodes can move with a certain speed to receive data packets only when they sojourn at certain parking positions. The movement trajectory mainly depends on the network periphery. Mobile sink nodes can move along the network periphery and cross the border in a certain area according to the actual layout.

Initially, there is only one mobile sink node existing in the sensor network, and it is located at one special point in the sensing field. On the basic of the value of the distance *d_sn_*, neighbor sensor nodes of the mobile sink node in each parking position are chosen for later use. If the value of the distance *d_sn_* is much smaller than the transmission radius, sensor nodes will transmit their monitored data to the sink node directly; otherwise, relay sensor nodes need to be elected to forward monitored data. Relay sensor nodes will be elected mainly based on the value of the link cost.

In order to improve communication efficiency and reduce long communication cost, a multi-hop communication manner is used in our proposed routing algorithm. If the distance *d_sn_* of sensor *s_i_* is larger than threshold *d_0_*, it needs to find an adjacent sensor as its relay node to forward its data. To achieve load balancing, relay nodes are chosen mainly based on the relative distance and the residual energy. In order to ensure a relay node with a better distance and enough energy is used, distance factor *D_f_* and energy factor *E_f_* metrics are defined in Equations (2) and (3), respectively, where *E(j)* is the residual energy of sensor nodes, and can get the maximum residual energy by flooding. Distance factor *D_f_* is related to the sum of the distance between source node *s_i_*, relay node *s_j_*, and destination node P_i_. Relay node will be chosen from the neighbor nodes of source node:

(2) $$D_{f}=\frac{d{\left(s_{i},s_{j}\right)}^{2}+d{\left(s_{j},{\text{P}}_{\text{i}}\right)}^{2}}{\max(d{\left(s_{i},s_{j}\right)}^{2}+d{\left(s_{j},{\text{P}}_{i}\right)}^{2})}$$

(3) $$E_{f}=\frac{\max(E(j))-E(j)}{\max(E(j))}$$

Link cost between sensor node *s_i_* and *s_j_* can be defined as is shown in Equation (4), where the value of *w* is between 0 and 1. If sensor node *s_i_* is far away from the current location *P_i_* of mobile sink nodes, it needs to choose a sensor node *s_j_* as its relay node. Sensor node *s_j_* with the minimum link cost will be chosen to relay monitored data:

(4) $$\text{link\_}\cos t(i,j)=(1-\omega )*D_{f}+\omega *E_{f}$$

Here, we define neighbor sensor nodes of sensor *s_i_* as those nodes existing in the transmission range of *s_i_* Thus neighbor sensor nodes of mobile sink nodes are elected based on the distribution of sensor nodes in rectangle network. Along the mobile sink node moving direction, neighbor sensor nodes with the longest distance will be defined as boundary sensor nodes. Thus, parking position *P_i_* can be determined mainly based on the transmission range of sensor nodes and boundary sensor nodes.

**Figure 2.**
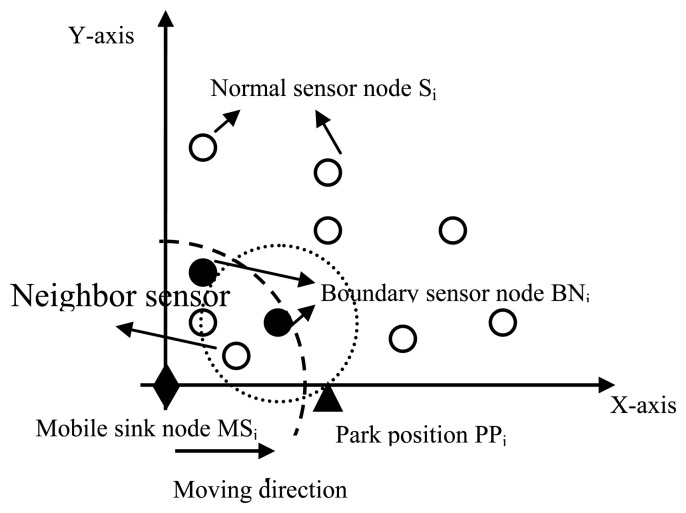
Parking position selection.

The selection of parking positions is shown in Figure 2. In this figure, we define the direction of X-axis as the direction of movement of mobile sink nodes, and mobile sink node *MS_i_* is located at the origin of coordinates. According to the value of the distance *d_sn_*, neighbor sensor nodes of *MS_i_* are chosen and shown as a black diamond in Figure 2. Boundary sensor nodes will be chosen from these neighbor nodes and marked with black dots. According to the transmission range of boundary sensor nodes, parking position *P_i_* can be determined and marked with a triangle. That is,, park positions are determined by the direction of movement and the transmission range of the boundary sensor nodes. If there is no neighbor sensor nodes of the mobile sink node located in a certain position, the mobile sink node will keep moving along the movement path for a certain distance until it finds a position with neighbor sensor nodes. The mobile distance depends on the actual situation. Mobile sink nodes will sojourn at each parking position for enough time to collect monitored data from the sensor nodes in a single-hop or multi-hop communication manner. The sojourn time is determined by the propagation speed and the maximum distance between the source nodes and the mobile sink node.

After completing the data transfer, the mobile sink node will move to the next parking position to receive data. While the mobile sink node moves from one parking position *P_i_* to another position *P_j_* it will not receive any monitored data from sensor nodes. When mobile sink node *MS_i_* moves to a parking position *P_i_*, it will broadcast a notification message, which includes an ARRIVAL_MSG message and a NEXT_ POSITION _MSG message.

**Figure 3.**
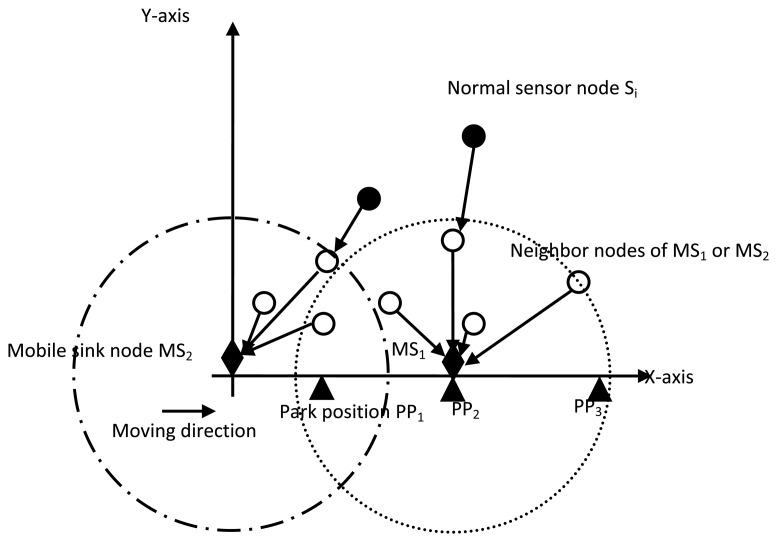
Multiple sink mobility strategy.

Each parking position will be kept in a table PP_TABLE for later use. This table will be saved in each mobile sink node, and updated before a new round of data transmission. When multiple mobile sink nodes exist in the rectangle network, we define that the starting point and speed of movement of each mobile sink node is the same. Each mobile sink node will move from the origin of coordinates in succession. The multiple sink mobility strategy is briefly described in Figure 3.

In Figure 3, at the point of departure, only one mobile sink node *MS_1_* is necessary to receive monitored data. After leaving the origin of coordinates, another mobile sink node *MS_2_* will participate in the data collection. Sojourn locations *SL_i_* of mobile sink nodes are all chosen from parking positions in the PP_TABLE. Each mobile sink node will chose one of the parking positions from the PP_TABLE as its park position to collect monitored data. Sensor nodes within the transmission range will communicate with mobile sink nodes directly, and will be responsible for other sensor nodes to forward data to each mobile sink node.

### 4.2. Route Steady Phase

In our proposed routing algorithm, sensor nodes monitor the sensing field and transmit their monitored data to their relay nodes and finally to the mobile sink node. To reduce energy consumption and improve data quality, data fusion can be used in each neighbor node of the mobile sink nodes.

On the basis of the nodes' distribution and transmission range, the selection of parking positions, which will be used for the mobile sink nodes to sojourn, was described in a previous section and the corresponding location information is kept in the table PP_TABLE.

**Figure 4.**
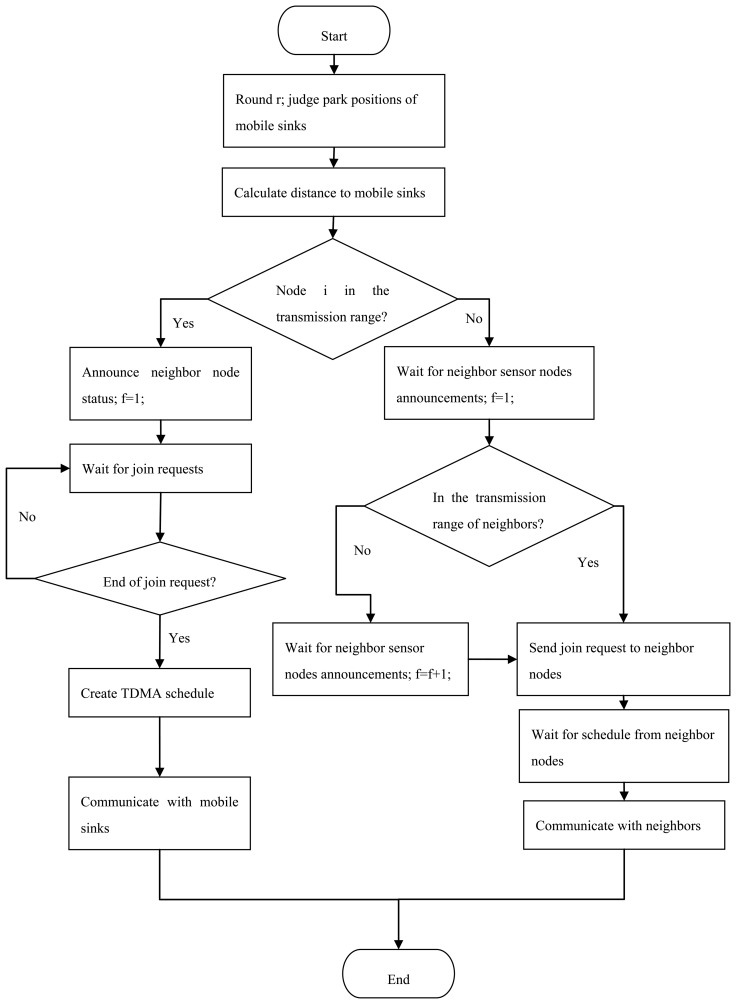
Flowchart of our proposed routing algorithm.

The process of our routing algorithm is described in Figure 4. In a certain round *r*, each mobile sink will select one parking position as its sojourn location to collect monitored data collaboratively. Then, node *i* will calculate the distance to the mobile sink to judge whether it is in the transmission range of mobile sink.

If yes, these neighbor sensor nodes will broadcast a NEIGHBOR_STATUS message, and wait for JOIN_REQUEST from the other sensor nodes. The neighbor sensor nodes of mobile sink are selected primarily based on the distance *d_sn_*. The neighbor sensors transmit monitored data to their mobile sink nodes directly, and they will keep awake to receive JOIN_REQUEST. A time division multiple access (TDMA) schedule is used to allocate transmission time slot for each neighboring sensor node, as is depicted in the left part of Figure 4.

If no, the mobile sink will wait for the next neighbor sensor nodes announcement as it moves to the next position. Then, it will repeat the process of judging its neighboring sensor nodes and sending JOIN_REQUEST messages, *etc.*, as isshown in the right part of Figure 4.

To reduce the energy consumption and guarantee network connectivity, each sensor can use power control to set its transmission power based on the distance to the receiver. The multi-hop route can be set up once all sensor nodes find relay sensor nodes. In order to avoid data packet collisions and reduce energy consumption, each neighbor sensor node of the mobile sink nodes will use a unique spreading code [3]. This unique spreading code will be sent from each mobile sink node to the corresponding next hop sensor nodes. That is, this code is spread to source sensor nodes from destination nodes. Thus each transmission path has a unique code and all sensor nodes will transmit their monitored data to neighbor sensor nodes using this spreading code. Data communication will be conducted in each link and the monitored data packets will be transmitted to mobile sink nodes in a single-hop or a multi-hop manner.

### 4.3. Route Maintenance Phase

If there were a transmission link failure in sensor network, the data communication in the corresponding transmission link will be truncated, causing network partition and isolated nodes. In other words, transmission link breaks have an important influence on data communication. Many factors can cause a link break. First, if any one sensor node hardware facility in the transmission link is damaged, it will cause the disruption in the corresponding transmission link. Second, the residual energy of each sensor node reduces gradually. The energy of a sensor node in the transmission link may be not enough to forward data packets, thus its corresponding transmission link will also be truncated, causing a certain area to be unreachable.

Therefore, considering the residual energy of each sensor nodes during the routing process is quite necessary to alleviate the transmission link break problem. In addition, other alternative forwarding sensor nodes need to be elected to replace the damaged sensor node or sensor node with insufficient energy to ensure complete data communication. Before data transmission in each round, sensor nodes will transmit a message including their ID, residual energy and status to mobile sink nodes.

**Figure 5.**
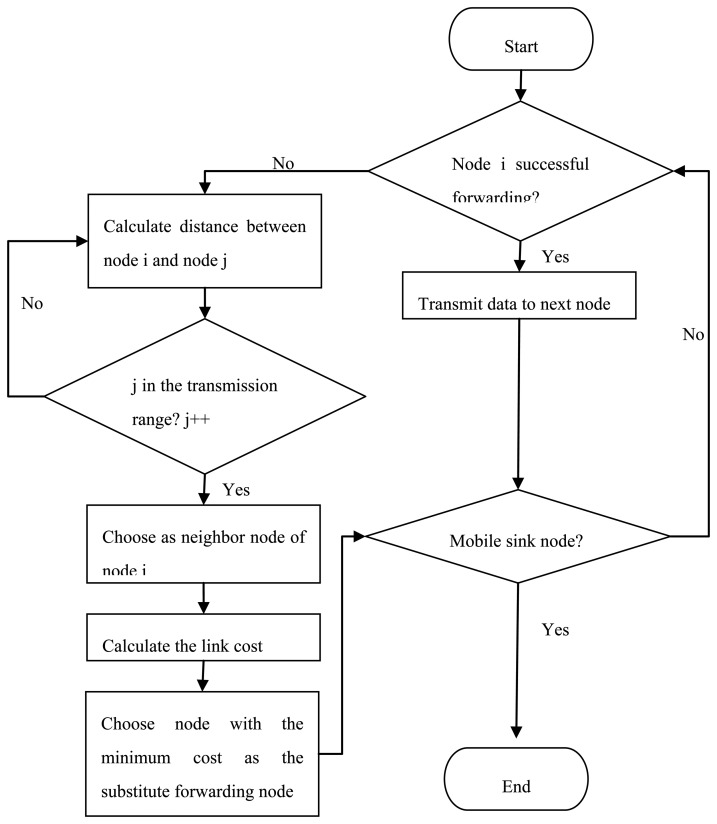
Flowchart to find a substitute forwarding sensor node.

The flowchart to find substitute forwarding sensor nodes is illustrated in Figure 5. If the residual energy of sensor node *s_i_* is not sufficient for the next data transmission, it will send a REJECT_MSG to its peripheral sensor node, and will not participate in the next data forwarding process. During the data communication process, if a sensor node *s_i_* in the transmission link has already failed, an adjacent sensor node near *s_i_* with the highest residual energy will be elected to act as the substitute forwarding sensor node. Thus we can ensure all sensor nodes in each transmission link can work properly and have enough energy to forward monitored data.

## 5. Performance Evaluation and Discussion

### 5.1. Simulation Environment

We use a Matlab simulator to evaluate the performance of our proposed routing algorithm here. In a 100 × 100 m^2^ rectangle sensor network, there are 100 sensor nodes randomly distributed. Initial energy *E_0_* of each sensor node is 2 Joules, and each sensor will generate a data packet of 2,000 bits. In order to guarantee a certain level of network connectivity, the transmission range of each sensor node is set to 50 to 80 m. Besides, other simulation parameters are defined as the follows: the energy dissipation *E_elec_* to run the radio device is set to 50 nJ/bit. The free space model of transmitter amplifier *ε_fs_* and the multi-path model of transmitter amplifier *ε_mp_* are respectively set to 10 pJ/bit/m^2^ and 0.0013 pJ/bit/m^4^.

### 5.2. Study on Mobile and Static Sink Cases

LEACH is a classical clustering algorithm which utilizes randomized rotation of local cluster head nodes to evenly distribute the energy load across the network [3,4]. Compared with other ordinary routing algorithms or protocols, it can prolong the network lifetime up to eight times. Here we compare the performance of our proposed algorithm with LEACH.

**Figure 6.**
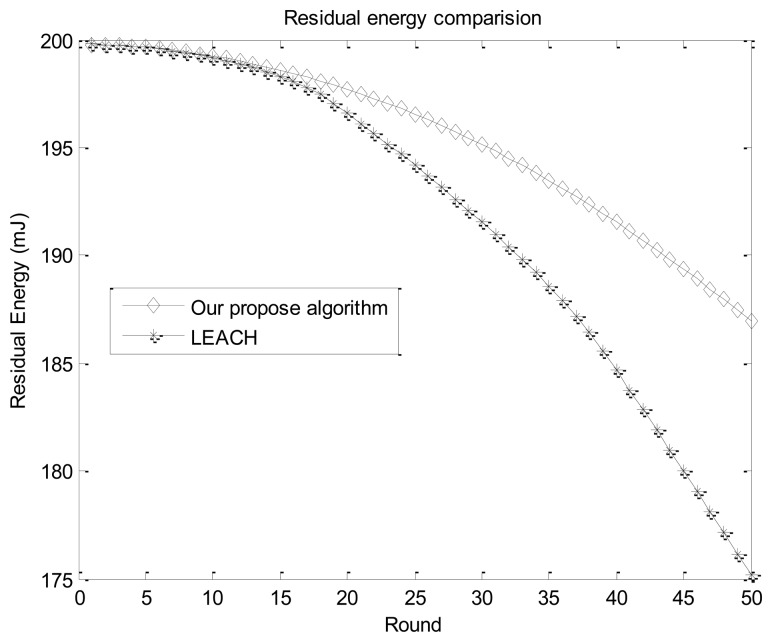
Residual energy comparison.

A comparison of the residual energy using multiple mobile sink nodes and LEACH is shown in Figure 6. We can find that energy consumption decreases with round (or time). The rate of decrease of LEACH is much faster than that of our proposed algorithm with multiple mobile sinks. The energy consumption rate is similar up to 17 rounds. After that, the energy consumption of our proposed algorithm is much less than that of LEACH. Consequently, the energy in LEACH network gets drained away much earlier, and our proposed routing algorithm outperforms LEACH.

### 5.3. Study on Mobile Sink Number

Much of the research on sink mobility technology has proven that the use of sink mobility technology can significantly improve sensor network performance. In addition, compared with mobile sensor nodes, mobile sink nodes are relatively easy to implement. Thus, we focus on studying the influence of multiple mobile sink nodes on energy consumption and network lifetime. In this paper, we mainly discuss the performance of a sensor network separately using three mobile sinks, two mobile sinks, and one mobile sink node.

**Figure 7.**
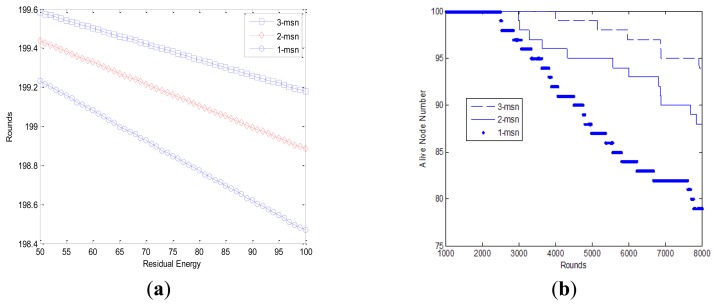
Simulation results with different mobile sink number. (**a**) Residual energy comparison; (**b**) Network lifetime comparison.

In Figure 7, “3-msn” represents that there are three mobile sink nodes working for the network. In Figure 7a, we compare the energy consumption of a sensor network with different numbers of mobile sink nodes. From Figure 7a, we can observe that the network residual energy performance is quite related with the number of mobile sink nodes. The performance of a sensor network using multiple mobile sink nodes is superior to that using a single mobile sink node. Network residual energy can be preserved with longer network lifetime at the cost of higher mobile sink numbers. From a practical application point of view, a trade-off is needed by considering both network performance and mobile sink cost.

The comparison of network lifetime factor using different numbers of mobile sink nodes is illustrated in Figure 7b. Network lifetime is defined as the time when the first sensor node depletes its energy. The vertical axis in Figure 7b represents the number of nodes remaining in the network. We can see that number of live sensors decreases sharply when there is only one mobile sink node, while the performance becomes better with more sink nodes.

Table 1 shows the specific round together with corresponding node ID when the first sensor node dies. We can find that three mobile sink nodes can best prolong the network lifetime than the other two scenarios, and the network lifetime is almost two times longer than the case with one sink node. However, the cost of mobile sink nodes is also higher than that of normal sensor nodes. Thus, we need to decide the number of mobile sink nodes according to the practical demand to achieve a trade-off between these factors.

### 5.4. Study on Mobile Sink Sojourn Location

We assume that mobile sink nodes are placed on public buses moving along the perimeter of the sensing field. Mobile sink nodes will start successively from the same position. Here, we analyze the performance of the sensor network with two mobile sink nodes in detail.

**Figure 8.**
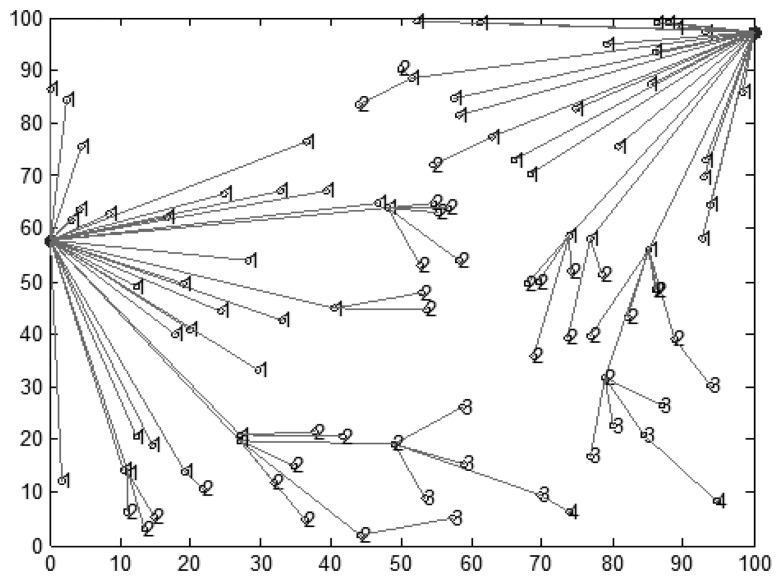
Network with two mobile sink nodes.

The network topology is shown in Figure 8, where sensor nodes communicate with mobile sink nodes in a multi-hop manner based on the value of the link cost. Mobile sink nodes move along the periphery of the rectangular region, and these sensor nodes can adjust their transmission range based on the value of *d_nn_* and the value of *d_sn_*. Here, *d_nn_* is the distance between sensor nodes, and *d_sn_* is the distance between sensor nodes and mobile sink nodes. According to the different value of *d_sn_*_,_ sensor nodes transmit data by multi-hop. Mobile sink nodes will stay at the special position long enough to collect data packets.

**Figure 9.**
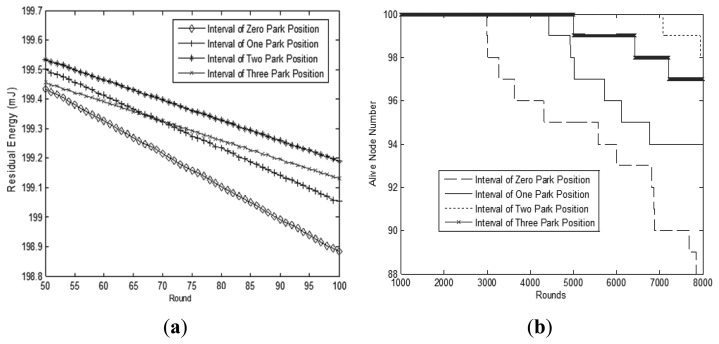
Simulation results with different sojourn location. (**a**) Residual energy comparison; (**b**) Network lifetime comparison.

Sojourn locations *SL_i_* are selected from parking positions which are determined in the previous experiments. Each mobile sink node needs to choose one parking position as its sojourn location to sojourn and wait for data collection. The influence on performance of the sensor network where mobile sink nodes chose different sojourn locations is shown in Figure 9.

In Figure 9a, we can observe the residual energy of the network over a period of time and find that the least energy is consumed when the interval of mobile sink nodes is two parking positions. Energy is consumed most when there is no interval between parking positions, which can be viewed as a continuous movement pattern. After 71 rounds, the performance using three intervals is superior to that using one interval between parking positions. Thus, we choose the two intervals to improve energy efficiency. In Figure 9b, we compare the network lifetime using different parking positions intervals. Each mobile sink node will chose different parking positions to sojourn in one round. Similarly, it can be observed that network performance with a two parking position interval is the best.

Table 2 shows the corresponding round and node ID when the first sensor node depletes its energy. The network lifetime is the longest when the mobile sink node interval is two parking positions. It is about two and half times the lifetime of the case when the sink node moves continuously. The first failed sensor node appears in the 7087^th^ rounds, so it lasts longer than the others. Thus we can conclude that when the interval of parking positions is set to 2, the network performance will be superior to the others.

## 6. Discussion

The introduction of multiple mobile sinks for WSNs can balance energy consumption, prolong network lifetime, and reduce sensor nodes' overhead near mobile sink nodes. Besides, they are well suited for sparse or disconnected sensor networks. They can also provide well guaranteed network connectivity. However, the cost of mobile sinks is relatively higher than that of normal sensor nodes. Thus, selection of an appropriate number of mobile sink nodes needs to be further studied under various applications to optimize the tradeoff between cost and network performance.

If sink nodes move along a predefined path in the sensor network, the predefined movement path and park positions need to be carefully designed. We assume that there is no big obstacle in the movement path, so that mobile sinks can move along the trajectory smoothly and sojourn at those park positions to collect monitored data from static sensor nodes in a multi-hop transmission manner.

This is a joint optimization problem of both sink moving strategy and routing algorithm, which is a NP-hard problem. As sink nodes are moving, the network topology is changing accordingly and route information will also get updated. The key problem is how to define relevant models like energy and traffic model to calculate network metrics like energy consumption and lifetime under different sink node(s) moving strategies or applications.

In our experiments, the number of mobile sink nodes is obtained by analyzing the performance with different mobile sink numbers. We analyze the performance of the network using one to three mobile sink nodes, respectively, and finally choose to deploy two mobile sink nodes for data collection. In addition, the sojourn location of each mobile sink node which is elected from parking positions is relatively hard to determine. In order to make the sensor network have better flexibility, parking positions are elected based on the X_axis or the Y_axis coordinate and transmission range of the boundary neighbor node in the moving direction, and sojourn locations will be chosen from these parking positions.

## 7. Conclusions

In this paper, we have proposed an energy efficient distance-aware routing algorithm with multiple mobile sinks for WSNs to improve network performance in terms of energy consumption and network lifetime. We first compare the residual energy of sensor network respectively using our proposed routing algorithm and LEACH, and find that mobile sink nodes clearly contribute to energy efficiency. Then we further study the network performance with different mobile sink numbers and different sojourn location selections. Through extensive experimental analysis, we see that mobile sink nodes can greatly improve energy efficiency and prolong network lifetime. In our proposed routing algorithm, sojourn location is chosen from parking positions determined by the nodes' distribution and transmission range. In order to ensure completeness and correctness of forwarded data packets, relay sensors are selected based on the distance factor and energy factor. Besides, if a certain transmission link fails, a substitute forwarding sensor node is selected depending on the link cost between sensor nodes.

In order to alleviate the hot spot problem and further balance the energy consumption, we use mobile sink nodes for data collection. However, all the experiments are conducted in an ideal environment, and it lacks a comparison of our proposed routing algorithm with some existing algorithms or protocols using mobile sink nodes. In this paper, we mainly put the emphasis on the study of the number of mobile sink nodes and parking position selection. The influence of different sink moving speeds on network performance is not analyzed. In the future, we will further study several known routing algorithms or protocols using multiple mobile sink nodes, and discuss the influence of sink moving speed and movement trajectory on packet delivery delay, energy consumption and network lifetime. Besides, to effectively organize and manage sensor nodes, we will study how to combine clustering technology with sink mobility technology so that network performance can be further improved.

## Figures and Tables

**Table 1.** Round when the first node dies.

**Network Environment**	**Node ID**	**Round**
Three mobile sink nodes	78	4004
Two mobile sink nodes	5	2994
One mobile sink node	98	2490

**Table 2.** Round when the first node dies.

**Network Environment**	**Node ID**	**Round**
Interval of zero parking position	5	2994
Interval of one parking position	5	4450
Interval of two parking positions	62	7087
Interval of three parking positions	28	5012
